# In situ single-cell profiling sheds light on *IFI27* localisation during SARS-CoV-2 infection

**DOI:** 10.1016/j.ebiom.2024.105016

**Published:** 2024-02-19

**Authors:** Chin Wee Tan, Jinjin Chen, Ning Liu, Dharmesh D. Bhuva, Tony Blick, James Monkman, Caroline Cooper, Malvika Kharbanda, Kristen Feher, Belinda Phipson, Emily E. Killingbeck, Liuliu Pan, Youngmi Kim, Yan Liang, Andy Nam, Michael Leon, Paulo Souza-Fonseca-Guimaraes, Seigo Nagashima, Ana Paula Camargo Martins, Cleber Machado-Souza, Lucia de Noronha, Benjamin Tang, Kirsty Short, John Fraser, Gabrielle T. Belz, Fernando Souza-Fonseca-Guimaraes, Arutha Kulasinghe, Melissa J. Davis

**Affiliations:** a Division of Bioinformatics, Walter and Eliza Hall Institute of Medical Research, Melbourne, VIC, 3052, Australia; b Department of Medical Biology, Faculty of Medicine, Dentistry and Health Sciences, University of Melbourne, Parkville, VIC, 3010, Australia; c Frazer Institute, Faculty of Medicine, The University of Queensland, Brisbane, QLD, Australia; d South Australian ImmunoGENomics Cancer Institute, The University of Adelaide, SA, Australia; e Princess Alexandra Hospital, Woolloongabba, QLD, Australia; f Nanostring Technologies, Inc, Seattle, WA, USA; g Pontifícia Universidade Católica do Paraná, PUCPR, Curitiba, Paraná, Brazil; h Laboratório de Patologia Experimental, PPGCS da PUCPR, Curitiba, Brazil; i Faculdades Pequeno Príncipe (FPP), Instituto de Pesquisa Pelé Pequeno Príncipe (IPPPP), R. Silva Jardim, 1632-ÁguaVerde, Curitiba, 80230-020, PR, Brazil; j Westmead Institute for Medical Research, Sydney, Australia; k School of Chemistry and Molecular Biosciences, The University of Queensland, Brisbane, Australia; l Critical Care Research Group, The Prince Charles Hospital, Brisbane, Australia; m Faculty of Medicine University of Queensland, Brisbane, QLD, Australia; n St Andrew's War Memorial Hospital, UnitingCare, Spring Hill, QLD, Australia; o Department of Clinical Pathology, Faculty of Medicine, Dentistry and Health Sciences, University of Melbourne, Parkville, VIC, 3010, Australia

## Abstract

The utilization of single-cell resolved spatial transcriptomics to delineate immune responses during SARS-CoV-2 infection was able to identify M1 macrophages to have elevated expression of IFI27 in areas of infection.

The SARS-CoV-2 pandemic has affected over 600 million people to date, resulting in over 6.8 million deaths, with a hospitalisation rate of approximately 20% and numerous long-term sequalae in patients.^1^ Acute respiratory distress syndrome (ARDS) occurs in 40% of patients infected with SARS-CoV-2 and can lead to pneumonia and death. The pathogenesis of ARDS is linked to inflammatory injury to the alveolar-capillary membrane, which can impair lung function. There is a growing need to understand the molecular mechanisms underpinning SARS-CoV-2 pathogenesis to aid in the prognosis and therapeutic management of SARS-CoV-2. This study aimed to analyse the individual cells present in SARS-CoV-2 rapid autopsy tissues by spatially resolved single-cell transcriptomic methods to localise *IFI27* expression levels. Our previous multi-cellular study identified that *IFI27* was highly expressed in the lung tissues of patients infected with SARS-CoV-2 when contrasted to non-viral infected tissues.^2^

We retrospectively analysed rapid autopsy lung tissues from 18 patients infected with SARS-CoV-2 who died from respiratory failure (ARDS), with SARS-CoV-2 infection confirmed by RTqPCR of nasopharyngeal swabs (Appendix pp 3, Table S1). Tissue blocks were reviewed by an anatomical pathologist and 2 tissue microarrays constructed from 30 representative cores, with up to 2 cores per patient. Adjacent serial tissue sections were profiled with RNAscope (ACDBio, USA) for SARS-Cov-2 and the CosMx™ Spatial Molecular Imager (SMI) 1000-plex assay (NanoString® Technologies, USA), as previously described^3^ (Appendix pp 3–4).

The dataset comprises 112 fields of views (FOVs) across 60 cores (2 sequential sections per TMA) with the expression matrices as integrated counts defined by cell segmentation provided by NanoString Technologies. Data were pre-processed based on pipeline previously established^4^ (Appendix pp 5–6). Briefly, low quality cells (<10% quantile) and FOVs (<200 cells per FOV) were removed. log 2-transformed counts per million (logCPM) were normalized using SCTransform (in Seurat R package) and cell type annotations established using a workflow implementing a majority consensus strategy of 4 widely used annotation methods for scRNAseq data; Azimuth, CelliD, singscore and AUCell, with 71% of the cell types annotated (spatially depicted in Fig. 1a). Using this consensus approach, we identified 25 cell type categories. The macrophages identified were further subclassified using 3 marker based methods (Singscore, CelliD and AUCell) using a unique set of marker genes collated from Azimuth's HLCA (Human–Lung v2 annotation level 4) and list published by Aegerter and colleagues^5^ (Fig. 1a).

**Fig. 1:**
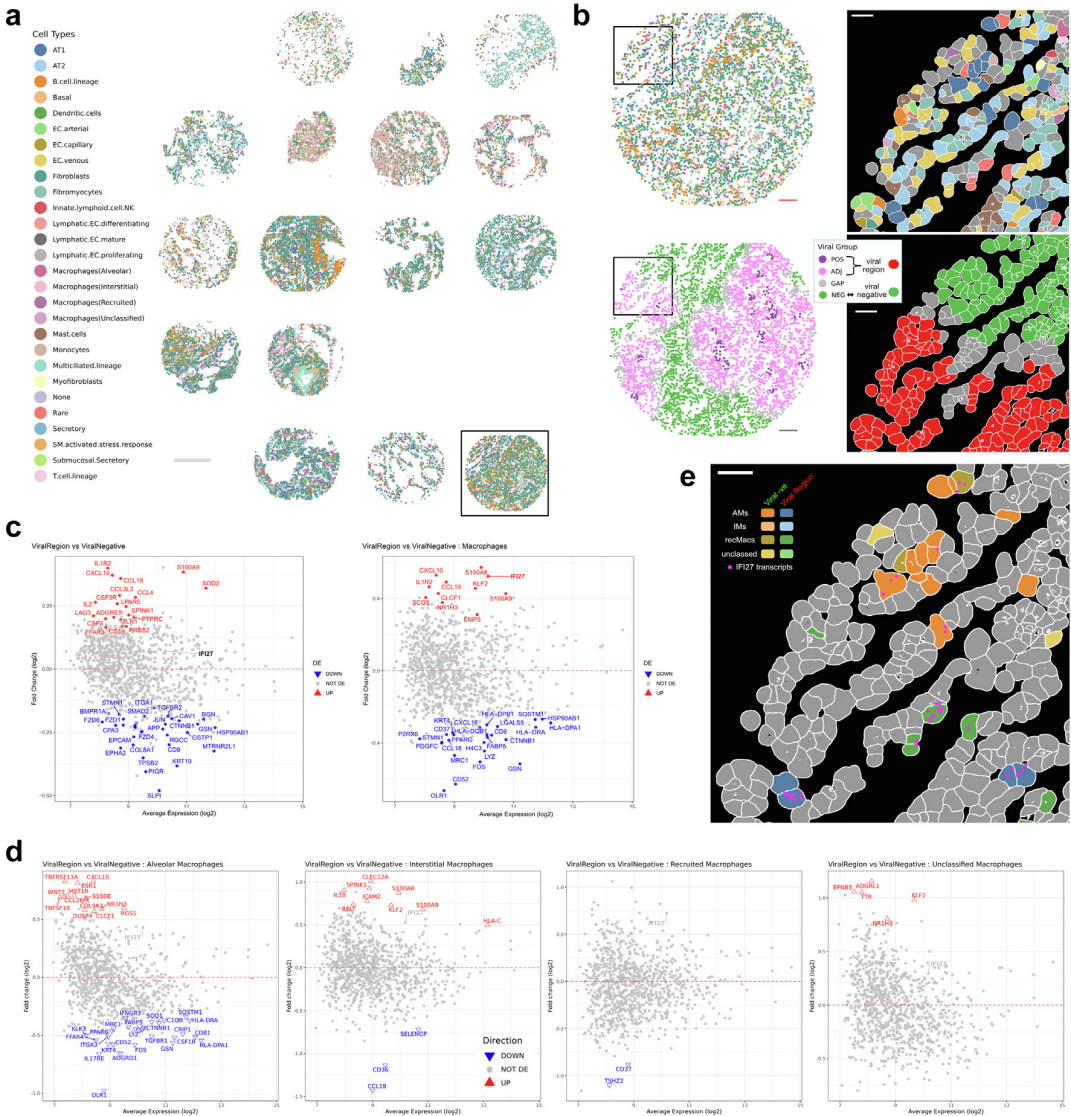
a) Spatial cell type mapping for one section of the two tissue microarrays used in the study showing the cell type distributions amongst and between the tissue cores. 28 categories of cell types were identified using a majority voting strategy using 4 cell annotation methods. Identified macrophages were further reannotated using an integrated marker lists in alveolar, interstitial, recruited or unclassified macrophages. b) Zoomed in spatial maps of the boxed core (left) and boxed region (right) showing the heterogeneous cell type neighbourhood (top) and viral groups (bottom) as defined based on alignment of RNAscope images of viral signal aligned with the CosMx SMI spatial image intensities. POS: viral positive cells, ADJ: viral adjacent cells, GAP: gap region cells between POS and ADJ, NEG: viral negative cells. Viral region used in the downstream analyses include POS + ADJ. c) Differential expression analysis was conducted on pseudo-bulked samples using the *voom-limma-duplicatecorrelation with sample weights* pipeline with empirical Bayes moderated t-statistic and Benjamini–Hochberg multiple testing adjustment (adjusted p-value <0.05). Differentially expressed genes were visualized as a function of fold change (log 2) against the average transcript expression (log 2) (MA-plot) for the following comparison: Viral Region versus Viral Negative samples for (left) all cells regardless of cell type and (right) Macrophages only. d) DE genes visualised as MA plots for Viral Region versus Viral Negative samples amongst the different macrophage subtypes, namely. e) Zoom spatial mapping of cells in the boxed region of (B) depicting the spatial locations of different subtypes of macrophage cells in either the Viral Region or in the Viral Negative region. IFI27 transcript of macrophages cells. The location and distribution of IFI27 transcripts in these macrophages are shown. Scale bars: (a) 500 μm, (b) 100 μm and 30 μm (zoomed regions) (e) 30 μm.

Spatial cell locations (by CosMx SMI) and SARS-CoV-2 viral regions (by RNAscope) were integrated to identify areas with viral presence (Appendix pp 6). Briefly, RNAscope images were rescaled and rotated to adjust for differences in pixel resolution and section orientation, FOV-equivalent regions identified, Otsu threshold-ed, black background inverted, and outliers removed. The binary masks were overlayed with the CosMx cell centroid images, and 4 groups of cells were defined: 1) *viral positive*: cell centroids within 18 μm of a positive viral pixel; 2) *viral adjacent*: >18 μm & ≤100 μm; 3) *gap*: >100 μm & < 140 μm; *viral negative*: ≥ 140 μm. *Viral positive* and *viral adjacent* cells were grouped as the “*viral region*” for downstream analyses (Fig. 1b).

We then measured the transcriptional differences between viral infected and uninfected regions (*viral region* vs *viral negative*) (Appendix pp 6–7). Computational analyses were conducted using a pseudo-bulked differential expression (DE) analysis approach with the sample annotations, groups and cell type annotations integrated. The samples were pseudo-bulked based on either A) “slide”, “patient” and “group” or B) “slide”, “patient”, “group” and “cell type”, resulting in n = 153 and n = 919 pseudo-samples respectively, after filtering out samples with less than 20 cells and applying gene level QC using *edgeR::filterByExpr*. For each category (A or B), differential expression analyses were conducted using a *voom-limma-duplicatecorrelation with sample weights* pipeline using the *edgeR::voomLmFit* function to fit a linear model with “slide” as a covariate, and to estimate the consensus correlation across patients and account for patient variation as a random effect.^6^^,^^7^ DE were conducted for the following comparisons: For A) Viral Region vs Viral Negative and for B) Viral Region vs Viral Negative for each cell type. An empirical Bayes moderated t-statistic was generated with multiple testing adjustment carried out using the Benjamini–Hochberg procedure to identify statistically significant genes (adjusted p < 0.05).

Using our approach, we detected a number of genes differentially expressed at FDR <0.05 for Viral Region vs Viral Negative across all cell types (53 genes, Fig. 1c left panel). These genes are broadly involved in inflammatory pathways. *IFI27* was found to be elevated in the viral regions when compared to the non-viral regions (albeit not significant) when comparing all the cells in the respective regions (Fig. 1c, left panel). However, when this was refined by measuring transcriptional profiles of individual cell types between regions, *IFI27* expression was only found to be significantly higher in macrophages residing in the viral region compared with non-viral region (Fig. 1c, right panel). Further interrogating the macrophage subtypes, we found that *IFI27* was elevated in the viral regions in all the subtypes (Fig. 1d and e), although not significant, it suggests a broader macrophage effect rather than a subtype specific influence. These data validate the findings of our multi-cellular spatial comparative study which showed higher *IFI27* expression levels in the lungs of patients with SARS-CoV-2 [2] and identified macrophages as a cell type that expressed elevated *IFI27* expression in response to SARS-CoV-2 infection. Other studies have also found that this *IFI27* associated interferon response appears to be an early triage biomarker for SARS-CoV-2 disease severity, is associated with innate responsiveness^8^ and demonstrate that blood levels of *IFI27* mRNA are prognostic for SARS-CoV-2 patient outcome. Indeed, *IFI27* has been found to provide the highest accuracy for discriminating between test-negative controls and test-positive individuals in delineating SARS-CoV-2 infection^9^ highlighting the strong relationship between lung damage and a potential diagnostic biomarker.

Our results suggest that blood *IFI27* have a great potential as a surrogate marker of lung macrophages and clearly reflects local immune response in the infected tissue. This will provide extremely useful information for any clinicians looking to modulate the local inflammatory responses in the lung. Indeed, this work is timely as there are currently no non-invasive methods to achieve this other than conducting daily lung biopsies which is neither safe nor practical clinically. The identification of macrophages as a source of elevated *IFI27* levels may allow for a more specific and powerful prognostic blood *IFI27* mRNA test.

This study is limited in that only SARS-CoV-2 lung tissues from patients who died were measured in the group. Other studies though have also identified *IFI27* as a marker of severe viral infection, such as with respiratory syncytial virus (RSV) infected preterm infants, where elevated *IFI27* blood levels associated with more severe disease, more frequent and longer periods of hospitalization, and more mechanical ventilation.^10^ Furthermore, *IFI27* was found to be an indicator of the severity of Enterovirus 71-induced hand foot and mouth disease, where it differed significantly in peripheral blood mononuclear cells between patients with mild and severe disease (PBMCs).^11^ While cell phenotyping and differential analysis was restricted by the use of the 1000-plex assay, we envisage that such limitations in the future will diminish as these assays approach whole transcriptome readout.

In conclusion, our study shows that macrophages have elevated expression of *IFI27* in areas of SARS-CoV-2 infection. Using a pseudo-bulk approach, the nuances in the cell types are missed, which this study demonstrates and shows the utility of single-cell resolved spatial transcriptomics to delineate immune responses during SARS-CoV-2 infection.

## Contributors

Conceptualization: AK, JF, GTB, BT, KS, FSFG, MJD. Data curation: TB, JM, CC, KF. Formal Analysis: CWT, JC, NL, DDB, MK. Funding acquisition: AK, MJD. Investigation: EEK, LP, YK, YL, AN, ML. Methodology: CWT, AK, MJD, TB, BP. Project administration: AK, MJD. Resources: PSDG, SN, APCM, CMS, LN. Software: CWT, NL, JC, MK, DDB. Supervision: AK, JF, GTB, BT, KS, FSFG, MJD. Visualization: CWT, NL, JC. Writing—original draft: CWT, TB, AK. Writing—review & editing: CWT, TB, AK. All authors read and approved the final version of the manuscript. CWT, JC, NL verified the underlying data.

## Ethics statement

The study was approved by the Pontificia Universidade Catolica do Parana PUCPR the National Commission for Research Ethics (3.944.734/2020), with ratification by the University of Queensland Human Research Ethics Committee. All methods followed relevant guidelines and regulations. Families permitted the post-mortem biopsies and provided written informed consent.

## Declaration of interests

EEK, LP, YK, YL, AN and ML are employees of Nanostring Technologies. KRS is a consultant for Sanofi, Roche and NovoNordisk. The opinions and data presented in this manuscript are of the authors and are independent of these relationships. Other co-authors have no conflict of interest to declare.
