# Supplementary material for: In situ single-cell profiling sheds light on IFI27 localisation during SARS-CoV-2 infection
Source: eBioMedicine. 2024 Feb 19;101:105016. doi: 10.1016/j.ebiom.2024.105016 (PMC10884333; doi:10.1016/j.ebiom.2024.105016)
Supplement: Supplementary Table S1 [file mmc1.docx]

Table S1 Clinical information of patients used in the study.

|  | **Gender** | **Age (years)** | **Ventilatory parameters** | | | | | | **Pronation maneuver  P/F Ratio** | **Length of stay on Mechanical Ventilation (days)** |  | **Relevant initial laboratory tests** | | | | | | | |  | **Laboratory tests 24 hours before death** | | | | | | | |  | **SARS-CoV-2 PCR (nasal swab)** | **Computed tomography chest at admission** | **Invasive procedure** |
| --- | --- | --- | --- | --- | --- | --- | --- | --- | --- | --- | --- | --- | --- | --- | --- | --- | --- | --- | --- | --- | --- | --- | --- | --- | --- | --- | --- | --- | --- | --- | --- | --- |
|  |  |  | **↓ PEEP, with ideal PEEP** | **Compliance (cmH2O)** | **Plateau pressure (cmH2O)** | **Driving pressure** | **FiO2** | **P/F Ratio** |  |  |  | **C-Reactive Protein (mg /L)** | **Globular volume (%)** | **Haemoglobin (g/dL)** | **D-dimer (µg/mL)** | **Total leukocytes # / band Cells # (%) Lymphocytes # (%)** | **Creatinine (mg / dL)** | **Blood platelets (/mm³)** | **Troponin (pg/mL)** |  | **C-Reactive Protein (mg /L)** | **Globular volume** | **Haemoglobin (g/dL)** | **Total leukocytes # / band Cells # (%) Lymphocytes # (%)** | **Blood platelets (/mm³)** | **Creatinine (mg / dL)** | **Troponin (pg/mL)** | **D-dimer (µg/mL)** |  |  |  |  |
| **CASE 1 (LN1)** | F | 87 | 10 | 52 | 24 | 14 | 60 | 126 | PR 191 | 6 |  | 313 | 24.80% | 8.7 | 2014  (4x ref) | 15,100 1208 (8%) 604 (4%) | 1.1 | 366,000 |  |  | 201 | 26.60% | 9.5 | 15,000 2400 (16%)  1200 (8%) | 239,000 | 2.65 |  |  |  | + | ***B*** | No |
| **CASE 2 (LN2)** | M | 53 | 8 | 65 | 19 | 11 | 50 | 132 | AR 270 | 10 |  | 146 | 38.10% | 14 | 425  (norm ref) | 11,000  550 (5%)  990 (9%) |  | 325,000 |  |  | 133 | 19.50% | 7.2 | 50,200 3012 (6%)  3514 (7%) | 318,000 |  |  | 7394 |  | + | ***A*** | Yes - ECMO |
| **CASE 3 (LN3)** | F | 85 | Not used   (Palliative care / spontaneous ventilation throughout hospitalization) | | | | | | Not used | 0 |  | 393 | 28.70% | 9.9 |  | 9,900 396 (4%) 1,782 (18%) |  | 282,000 |  |  | 139.9 | 30% | 10 | 9,900 693 (7%)  891 (9%) | 481,000 |  |  |  |  | + | ***H*** | No |
| **CASE 4 (LN4)** | M | 73 | 8 | 37 | 22 | 14 | 40 | 260 | Not used | 10 |  | 83 | 25% | 8.6 | 3436 (>3x ref value) | 9,200 1,932(21%)  552 (6%) |  | 38,000 |  |  | 270 | 23% | 8 | 22,000 2420 (11%)  440 (2%) | 356,000 |  |  |  |  | + | ***A*** | Haemodialysis  3x a week |
| **CASE 6 (LN6)** | M | 80 | 10 | 55 | 20 | 10 | 70 | 170 | PR  225 | 21 |  | 52 | 37.50% | 13 | 816  (ref value 500) | 4,700 188 (4%) 423 (9%) |  | 112,000 | 10.9 |  | 407 | 29.40% | 9.7 | 9,400 1,034 (11%)  1,316 (14%) | 142,000 |  | 32.7 | 7394 |  | + | ***A*** | Tracheostomy |
| **CASE 7 (LN7)** | M | 81 | 12 | 52 | 18 | 6 | 45 | 237 | PR  192 | 8 |  | 301.6 | 31.20% | 11 | 13,662  (6x ref value) | 11,800 1,770 (15%) 354 (3%). |  | 252,000 | 149.5 |  | 291.5 | 27.10% | 9.1 | 24,900 1,743 (7%)  498 (2%) | 175,000 |  |  |  |  | + | ***A*** | Peritoneal Dialysis (daily) |
| **CASE 8 (LN8)** | F | 70 | 12 | 14 | 21 | 9 | 60 | 127 | PR 175 | 14 |  | 16 | 40.40% | 13 | 4,160^~^ | 7,380 5,850(80.6%)  820 (11.1%) | 1.77 | 218,000 | 10 ^^^ |  | 16 | 25.70% | 7.6 | 15,880  11,910 (75%) 1260 (7.9%) | NA | 0.89 | 278.9 | 1,129 |  | + | ***D + G*** + Cardiomegaly | No |
| **CASE 9 (LN9)** | M | 86 | 8 | 52 | 21 | 13 | 45 | 263 | Not used | 3 |  | 105.9 | 32.50% | 12 | 11,184^~^ | 5,900 708 (12%) 472 (8%) |  | 110,000 | 21.7 ^^^ |  | 307.5 | 25.20% | 9 | 8,900 445 (5%)  712 (8%) | 99,000 |  | 29 | 13,535 |  | + | ***D*** | No |
| **CASE 13 (LN13)** | F | 75 | 8 | 32 | 21 | 13 | 60 | 105 | PR -   192 | 8 |  | 300.8 | 30.10% | 11 | 1,652^~^ | 21,300 1,278 (6%)  1,917 (9%) | 8.22 | 205,000 | 39.5 ^α^ |  | 186.7 | 26.60% | 9.3 | 34,900 6,282 (18%)  2,094 (6%) | 155,000 | 3.24 | 14.5 |  |  | + | ***D + G***. Cardiomegaly. Presence of pulmonary consolidation, air bronchograms, and small pleural effusion. | Haemodialysis |
| **CASE 16 (LN16)** | M | 57 | 12 | 67 | 24 | 12 | 45 | 126 | AR 200 | 9 |  | 154.2 | 38.30% | 14 | 628^~^ | 14,500 1305 (9%) 435 (3%) | 0.82 | 186,000 | 5.0 ^^^ |  | 267.2 | 27% | 9 | 15,300 459 (3%)  2448 (16%) | 216,000 | 2.43 | 19.5 | 6,571 |  | + | ***E + F*** | Chemical Thrombolysis |
| **CASE 17 (LN17)** | F | 81 | 8 | 45 | 22 | 14 | 40 | 254 | Not used | 14 |  | 199.4 | 41.30% | 14 | 83,143^~^ | 22,100 1326 (6%) 663 (3%) | 1.33 | 243,000 | 42.1 ^α^ |  | 16.3 | 29.20% | 9.5 | 28,900 867 (3%)  1156 (4%) | 141,000 | 1.06 | 324.7 | 19,137 |  | + | ***B*** | Tracheostomy Chest Tube Left Pneumothorax |
| **CASE 18 (LN18)** | M | 57 | 12 | 39 | 26 | 14 | 50 | 90 | AR 162 | 36 |  | 47.7 | 43.10% | 16 | 431^~^ | 7,400 0 (0%) 1332 (18%) | 1.23 | 146,000 | 5.2 ^^^ |  | 281.6 | 23.10% | 7.8 | 15,800 6004 (38%)  1264 (8%) | 395,000 | 3.53 | 39.3**^*^** | 2,190^*^ |  | + | ***B*** | Tracheostomy |
| **CASE 20 (LN20)** | M | 75 | 8 | 35 | 20 | 12 | 95 | 90 | AR 286 | 9 |  | 267 | 43.10% | 15 | 152,174^~^ | 13,100 655 (5%) 917 (7%) | 1.61 | 200,000 | 13.3 ^^^ |  | 226.8 | 19.70% | 11 | 19,100 191 (1%)  573 (3%) | 336,000 | 2.14 |  |  |  | + | ***B + F.*** Presence of diffuse bilateral bronchiectasis. Presence of parasseptal emphysema. | Chest Tube Right |
| **CASE 21 (LN21)** | M | 70 | 10 | 30 | 26 | 16 | 100 | 86 | AR 265 | 15 |  | 156.9 | 52.80% | 18 | 1,848^~^ | 16,000 160 (1%) 320 (2%) | 1.37 | 160,000 | 1750.2^^^ |  | 8.7 | 32% | 11 | 10,500 525 (5%)  420 (4%) | 67,000 | 1.66 |  |  |  | + | ***E***. Interstitial Pulmonary Fibrosis. Cardiomegaly. Increased Pulmonary Artery Diameter (32mm) | Tracheostomy |
| **CASE 28 (LN28)** | M | 86 | 8 | 37 | 20 | 12 | 60 | 134 | Yes but NR | 2 |  | 86 | 36.90% | 14 | 848^~^ | 5,300 106 (2%) 1,060 (20%) | 1.61 | 122,000 | 6.7 ^^^ |  | 180 | 39.50% | 14 | 4,700 141 (3%)  282 (6%) | 132,000 | 2.05 | 43.9 | 1,857 |  | + | ***B*** | No |
| **CASE 29 (LN29)** | M | 83 | 10 | 50 | 20 | 10 | 60 | 128 | AR 312 | 13 |  | 147.3 | 38% | 14 | 1.010^~^ | 9,200 276 (3%)  184 (2%) | 1.26 | 190,000 | 13.2 ^^^ |  | 30.7 | 38.40% | 13 | 23,300 2,097 (9%)  233 (1%) | 436,000 | 1.67 | 64.2 | 1,857 |  | + | ***C*** | No |
| **CASE 30 (LN30)** | F | 72 | 10 | 40 | 24 | 14 | 100 | 122 | PR 175 | 28 |  | 111.6 | 34.10% | 12 | 1.010^~^ | 6,000 360 (6%)  1,020 (17%) | 0.75 | 222,000 | 6 ^^^ |  | 339.9 | 23.70% | 8.5 | 32,200 7,084 (22%)  1,288 (4%) | 190,000 | 2.96 | 33 | 5,058 |  | + | ***C*** | Tracheostomy Haemodialysis |
| **CASE 31 (LN31)** | F | 72 | 10 | 21 | 23 | 13 | 60 | 126 | PR 178 | 20 |  | 114 | 30.90% | 12 | 1.975^~^ | 5,900 295 (5%)  472 (8%) | 1.07 | 227,000 | 7.2 ^^^ |  | 53.9 | 21.20% | 7.7 | 7,900 474 (6%)  474 (6%) | 180,000 | 1.53 | 669.2 | 7,550 |  | + | ***C*** | Tracheostomy Haemodialysis |
|  | | | | | | | | | | | | | | | | |  |  |  |  |  |  |  |  |  |  |  |  |  |  |  |  |
| *PaO2 : Partial Pressure of Oxygen* | | | | | | | | | *^~^ ref value <500* | | | | | | ***A*** | *Diffuse and bilateral “opacities with ground-glass attenuation”, suggestive of viral pulmonary infection* | | | | | | | | | | | | | | | |  |
| *P/F Ratio : PaO2 / FiO2* | | | | | | | | | *^^^ ref value <19.8* | | | | | | ***B*** | *Diffuse and bilateral “opacities with ground-glass attenuation”, thickening of the pulmonary septum, suggestive of viral pulmonary infection.* | | | | | | | | | | | | | | | | |
| *ECMO : Extracorporeal Membrane Oxygenation* | | | | | | | | | *^*^ 72 hours before death* | | | | | | ***C*** | *Diffuse and bilateral “opacities with ground-glass attenuation”, thickening of the pulmonary septum, suggestive of viral pulmonary infection, with involvement more than 50% of the lung parenchyma.* | | | | | | | | | | | | | | | | |
|  | | | | | | | | | *^α^ ref value <11.6* | | | | | | ***D*** | *Peripheral, multifocal and bilateral “opacities with ground-glass attenuation”, thickening of the pulmonary septum, suggestive of viral pulmonary infection* | | | | | | | | | | | | | | | | |
| ***Response to pronation maneuver*** | | | | | | | | |  | | | | | | ***E*** | *Peripheral, multifocal and bilateral “opacities with ground-glass attenuation”, suggestive of viral pulmonary infection* | | | | | | | | | | | | | | | |  |
| *PR: Partial response* | | | | | | | | |  | | | | | | ***F*** | *Presence of bronchial thickening.* | | | | |  |  |  |  |  |  |  |  |  |  |  |  |
| *AR: Adequate response* | | | | | | | | |  | | | | | | ***G*** | *Presence of centrilobular and parasseptal emphysema* | | | | | | | | |  |  |  |  |  |  |  |  |
| *NR: No response* | | | | | | | | |  | | | | | | ***H*** | *Image of “opacities with ground-glass attenuation” upper left lobe, suggestive of viral pulmonary infection, and small volume pleural effusion* | | | | | | | | | | | | | | | | |
