# Supplementary material for: In situ single-cell profiling sheds light on IFI27 localisation during SARS-CoV-2 infection
Source: eBioMedicine. 2024 Feb 19;101:105016. doi: 10.1016/j.ebiom.2024.105016 (PMC10884333; doi:10.1016/j.ebiom.2024.105016)
Supplement: Supplementary [file mmc2.docx]

**Supplementary appendix**

Supplement to: Chin Wee T, Jinjin C, Ning L … et al. In situ single-cell profiling sheds light on IFI27 localisation during SARS-CoV-2 infection

**Supplementary Appendix**

TABLE OF CONTENTS

[Materials and Methods 3](#_Toc157534320)

[Patient cohort 3](#_Toc157534321)

[RNAscope (SARS-CoV-2) 3](#_Toc157534322)

[Nanostring CosMx™ Spatial Molecular Imager (SMI) sample preparation 3](#_Toc157534323)

[CosMx SMI instrument run 4](#_Toc157534324)

[Data processing and quality control 5](#_Toc157534325)

[Cell type annotation 5](#_Toc157534326)

[Identifying viral load and regions 6](#_Toc157534327)

[Bioinformatics analyses 6](#_Toc157534328)

[Data exploratory and pseudo-bulking samples: 6](#_Toc157534329)

[Quality control and normalization 7](#_Toc157534330)

[Differential expression analysis 7](#_Toc157534331)

[Statistics 7](#_Toc157534332)

[Data Availability 7](#_Toc157534333)

[Roles of Funders 7](#_Toc157534334)

[Supplementary References: 8](#_Toc157534335)

Materials and Methods

Patient cohort

Autopsy and biopsy materials were obtained from the Hospital Marcelino Champagnat in Curitiba, Brazil, in accordance with the National Commission for Research Ethics (CONEP) under Pontificia Universidade Catolica do Parana PUCPR ethics approval numbers: protocol number 3.944.734/2020 approved under University of Queensland Human Research Ethics Committee ratification. Families permitted the post-mortem biopsy of COVID-19 samples. The study was All SARS-CoV-2 infected patients were confirmed for infection through RTqPCR of nasopharyngeal swabs specimens, and imaging with computed tomography (CT) showed diffuse and bilateral opacities with ground-glass attenuation, consistent with viral pulmonary infection. Radiographically-evident and representative lesions were sampled through an anterior mini-thoracotomy on the fourth or fifth intercostal space. Elapsed time between patient death and completion of the mini-thoracotomy after obtaining the consent form from the relatives did not exceed 4 hours. The samples were then fixed and kept in 10% formalin for at least 24 hours prior to blocking and slicing for microscopic analysis.

Sections from 19 patients were stained by H&E and reviewed by an anatomical pathologist to select representative tissue regions for TMA generation. Duplicate 1mm regions from each sample were cored and mounted into separate TMAs (TMA “A” and TMA “B”). 5µm serial sections of each TMA were mounted onto the reverse side of Leica Bond Plus slides, prior to RNAscope and the Nanostring CosMx Spatial Molecular Imager workflows. Clinical data were obtained from medical records during hospitalisation in the ICU (at Hospital Marcelino Champagnat in Curitiba, Brazil) - Table S1.

Although the TMAs were generated from 19 cases, the tissue for case LN13 (which had clinical information) was lost during processing while for another case (LN12), there are no clinical information available but was included in the analysis.

Adjacent serial tissue sections were profiled with RNAscope (ACDBio, USA) for SARS-Cov-2 and the CosMx™ Spatial Molecular Imager (SMI) 1000-plex assay (NanoString® Technologies, USA).

RNAscope (SARS-CoV-2)

RNAscope® probes (ACDbio, US) targeting SARS-CoV-2 spike mRNA (nCoV2019, #848561-C3) were used as per manufacturer instructions for automation on Leica Bond RX. DNA was visualised with Syto13 (Thermofisher Scientific), channel 1 with Opal 570 (1:500), channel 2 with Opal 620 (1:1500), and channel 3 with Opal 690 (1:1500) (PerkinElmer). Fluorescent images were acquired with Nanostring Mars prototype DSP at 20x.

Nanostring CosMx™ Spatial Molecular Imager (SMI) sample preparation

Formalin-fixed, paraffin-embedded (FFPE) tissue sections were prepared for CosMx™ Spatial Molecular Imager (SMI) profiling as previously described (1). Briefly, five-micron tissue sections on VWR Superfrost Plus Micro slides (cat# 48311-703) were baked overnight at 60°C, then prepared for in-situ hybridization (ISH) by deparaffinization and heat-induced epitope retrieval (HIER) at 100°C for 15 minutes using ER1 epitope retrieval buffer (Leica Biosystems product, citrate-based, pH 6.0) in a pressure cooker.

Following HIER, tissue sections were digested with 3 µg/ml Proteinase K diluted in ACD Protease Plus at 40°C for 30 minutes. Tissue sections were washed twice with diethyl pyrocarbonate (DEPC)-treated water (DEPC H2O) and incubated in 1:2,000 diluted fiducials (Bangs Laboratory) in 2X SSCT (2X saline sodium citrate, 0.001% Tween-20) solution for 5 min at room temperature in the dark. Excess fiducials were rinsed from the slides with 1X phosphate buffered saline (PBS) and tissue sections were fixed with 10% neutral buffered formalin (NBF) for 5 min at room temperature. Fixed samples were rinsed twice with Tris-glycine buffer (0.1M glycine, 0.1M Tris-base in DEPC H2O) and once with 1X PBS for 5 min each before blocking with 100 mM N-succinimidyl (acetylthio) acetate (NHS-acetate, ThermoFisher) in NHS-acetate buffer (0.1M NaP, 0.1% Tween PH 8 in DEPC H2O) for 15 min at room temperature. The sections were then rinsed with 2X saline sodium citrate (SSC) for 5 min and an Adhesive SecureSeal Hybridization Chamber (Grace Bio-Labs) was placed over the tissue.

NanoString® ISH probes were prepared by incubation at 95°C for 2 min and placed on ice, and the ISH probe mix (1nM ISH probes, 1X Buffer R, 0.1 U/μL SUPERase•In™ [Thermofisher] in DEPC H2O) was pipetted into the hybridization chamber. The hybridization chamber was sealed to prevent evaporation, and hybridization was performed at 37°C overnight. Tissue sections were rinsed of excess probes in 2X SSCT for 1 min and washed twice in 50% formamide (VWR) in 2X SSC at 37°C for 25 min, then twice with 2X SSC for 2 min at room temperature and blocked with 100 mM NHS-acetate in the dark for 15 min. A custom-made flow cell was affixed to the slide in preparation for loading onto the CosMx SMI instrument.

CosMx SMI instrument run

RNA target readout on the CosMx SMI instrument was performed as described (1). Briefly, the assembled flow cell was loaded onto the instrument and Reporter Wash Buffer was flowed to remove air bubbles. A preview scan of the entire flow cell was taken, and 15-25 fields of view (FOVs) were placed on the tissue to match regions of interest identified by H&E staining of an adjacent serial section. RNA readout began by flowing 100 μl of Reporter Pool 1 into the flow cell and incubation for 15 min. Reporter Wash Buffer (1 mL) was flowed to wash unbound reporter probes, and Imaging Buffer was added to the flow cell for imaging. Nine Z-stack images (0.8 μm step size) for each FOV were acquired, and photocleavable linkers on the fluorophores of the reporter probes were released by UV illumination and washed with Strip Wash buffer. The fluidic and imaging procedure was repeated for the 16 reporter pools, and the 16 rounds of reporter hybridization-imaging were repeated multiple times to increase RNA detection sensitivity.

After RNA readout, the tissue samples were incubated with a 4-fluorophore-conjugated antibody cocktail against CD298/B2M (488 nm), PanCK (532 nm), CD45 (594 nm), and CD3 (647 nm) proteins and DAPI stain in the CosMx SMI instrument for 2 h. After unbound antibodies and DAPI stain were washed with Reporter Wash Buffer, Imaging Buffer was added to the flow cell and nine Z-stack images for the 5 channels (4 antibodies and DAPI) were captured.

Data processing and quality control

Data used in this study is generated by CosMx™ Spatial Molecular Imager (SMI) profiling with NanoString’s SMI 1000 plex Human gene panel (v1). The data were measurements of RNA abundance of 980 genes, and 20 negative control (NegProbe) probes. Transcriptomic measurements were made within the regions of interests of each core at the sub-cellular level.

The SARS-CoV-2 lung SMI data consist of 4 tissue microarrays (TMA-A-8, A-9, B-9 and B-10), each with between 12 to 17 cores. TMA-A-8 and TMA-A-9 are consecutive sections of the respective core (i.e. matching), as is the case for TMA-B-9 and TMA-B-10. Each core has 1 to 2 FOVs capturing the whole area. A total of 112 FOVs across 60 cores were analysed with factors of consideration including patient of origin, SARS-CoV-2 viral load and cell type.

In this study, the pre-processing and quality control of the data was done based on pipeline previously established (2) for spatial transcriptomics which is briefly described as follows: the raw data is the expression counts of every gene in each cell. These expression matrices (from all TMAs) are integrated counts based on cells defined by the cell segmentation strategy provided by Nanostring (1). The matrices and metadata for the genes and cells (i.e. id and spatial coordinates) are then incorporated as a *SpatialExperiment* object in R for analysis. Quality control (QC) was performed on this cell level data, with cell level QC filtering out cells with total transcript count less than 10% of the quantile of the data (low quality cells). After removing the low-quality cells, fields of view (FOV) based quality control was conducted analysing both the library size and cell count per FOV to filter out low-quality FOVs. Visual inspection of the FOV distributions led to a 200 cells per FOV cutoff being used in this study. The rescaled log2-transformed counts per million (logCPM) of the data is provided as inputs for cell type annotation in the following section.

Cell type annotation

Cell type annotations of the data were determined using a customized workflow that implements a majority consensus strategy of four widely used annotation methods for scRNAseq data. The 4 methods include reference-based methods Azimuth (3) and CelliD (4) as well as marker-based methods singscore (5) and AUCell (6). For the reference-based methods, the core consensus reference model of the cellular landscape of the lung and nose from Human Lung Cell Atlas (HLCA) (7) was used as the reference. This reference includes 107 individuals from 14 datasets with 584,884 cells. For marker-based methods, cell type specific marker lists were determined by integrating CIBERSORTx (8) and HDBSCAN (a density based hierarchical clustering algorithm) (9). Here, we generated signature expression matrix from HLCA for genes in the dataset of interest using CIBERSORTx which is passed to HDBSCAN for clustering of cell types. Normalized expression of each marker for every cell type in the reference generated by CIBERSORTx is clustered by HDBSCAN to find the extreme group of “outlier” cell types with high expression. Each marker would proceed to be assigned to the cell types that’s being identified in the specific “outlier” group. The resulting marker lists were validated using the R package Garnett (10) and assessed by expert immunologist Dr Joanna Groom from WEHI.

For consistency, both the filtered counts data from the quality control step (logCPM) and data from the query dataset (HLCA) were normalized using the *SCTransform* function in the *Seurat* (3) R package. The normalized data was passed to each of the 4 methods to generate the respective cell type labels. The final cell type annotation was determined by a majority consensus of at least 2 out of 4 methods. This provides a reasonably good coverage of the cell types with 71.12%.

The macrophages identified were extracted and further subclassified using 3 marker-based methods (singscore, CelliD and AUCell) using a unique set of marker genes collated from Azimuth’s HLCA (Human - Lung v2 annotation level 4) and the curated marker list published by Aegerter and colleagues(11). This subclassification was then integrated with the other cell annotations for downstream analyses.

Identifying viral load and regions

To integrate spatial location, transcript information and covid-19 viral load, we employed a computational realignment strategy that uses the RNAscope images of the matching consecutive section of each core to identify cells in the cosMX data which have “high” viral load. Matching consecutive sections are utilized to ensure close correspondence and as accurate an indication of viral signal.

Firstly, as the RNAscope images and SMI images were acquired on different instruments on consecutive serial sections, to map the position on the RNAscope images onto the SMI acquired image, the RNAscope images are realigned computationally using matlab using a transformation and translation functions. The realignments are validated visually using known landmarks/features on the adjacent sections.

Secondly, binary masks of the realigned RNAscope images were generated. For this, an imageJ batch script was implemented that does the following steps: (1) Flip the aligned RNAscope image vertically and (2) apply Otsu thresholding using auto adjusted threshold values for each TMA (TMA A: 29419 of 65535, TMA B: 5000 of 65535). (3) invert image to black background, (4), binarize image and remove outliers (TMA A: radius = 3, TMA B: radius = 7, threshold =50, bright pixels). The final images are (5) flipped vertically and (6) saved as tiffs.

Thirdly, the binary mask images were loaded into R, flipped vertically, and converted to PNG file format. The positions of the centroids of each of the cells identified in the cosMX dataset were loaded and overlayed with the image mask. Based on this overlayed image, 4 groups of cells were defined:

1) *viral positive* cells: cell centroids within 18µm of a positive pixel.

2) *viral adjacent* cells: cell centroids >18µm and ≤ 100µm of a positive pixel.

3) *gap* cells: cell centroids >100µm and < 140µm of a positive pixel.

4) *viral negative cells*: cell centroids ≥140µm of a positive pixel.

For downstream analyses, *viral positive* and *viral adjacent* cells were grouped together as the “*viral region*”.

Bioinformatics analyses

Data exploratory and pseudo-bulking samples: The viral load cell groupings, sample annotations and cell annotations were integrated for further analysis. Inspection of the dataset and correlations between the cell types, slides, viral cell groups and spatial locations were conducted. The FOVs (or samples) were then pseudo-bulked based on either ***A***) “slide”, “patient” and “group” or ***B***) “slide”, “patient”, “group” and “cell type”. After filtering out samples with less than 20 cells and applying gene level QC using *edgeR::filterByExpr*, the pseudo-bulked samples consist of n=153 and n=919 pseudo-samples respectively.

Quality control and normalization*:* QC and normalization were conducted based on the *standR* workflow (2). Briefly, relative log expression (RLE) plots were used to assess the presence of any unwanted variation and/or batch effects (12). The pseudo-bulked data were then normalized using the trimmed mean of M-values (TMM) method (13) using all the genes in the panel. In brief, log2-transformed abundance data were median-centred first for each gene, and then within each sample. The difference between the observed and the population median of each gene was calculated. Principal components analysis (PCA) of the pseudo-samples was then utilized to identify variability related to particular factors in the dataset or experimental design.

Differential expression analysis*:* Differential expression (DE) analysis was performed using R packages *edgeR* (14) (v3.38.4) and *limma* (15) (3.52.4). Very briefly, DE was modelled using linear models with experimental factors as predictors. The variation in gene expression was modelled as a combination of the common dispersion that applies to all genes and the gene specific dispersion. To estimate the common and gene wise variation, an empirical Bayes approach was used to model the variation of each gene, borrowing information from all other genes while treating patients’ variation as a random effect using the *DuplicateCorrelation* function in *limma*. The linear model was then fitted to an experimental design and specific contrasts of interest applied to query the data for differential expression. This results in a more robust empirical Bayes moderated t-statistic compared with a t-statistic from a classic t-test. The Benjamini–Hochberg procedure was applied for multiple testing adjustments, with an adjusted p < 0.05 used to define significant DE genes. In this study, the *edgeR::voomLmFit* function was used to fit a linear model with “slide” as a covariate and the main factors of interest investigated include for ***A***) Viral Region vs Viral Negative and for ***B***) Viral Region vs Viral Negative for each cell type.

Statistics

The linear modelling conducted in the study uses a robust empirical Bayes moderated t-statistic and the Benjamini–Hochberg procedure was applied for multiple testing adjustments (this study uses an adjusted p-value of < 0.05).

Data Availability

The data underpinning this study is available for sharing. Following the publication of this work, the raw processed data for the NanoString cosMx dataset will be made available on Gene Expression Omnibus (GEO) with accession number GSE253474. High-resolution image files will only be made available upon reasonable request.

Roles of Funders

The funders of this study do not have any role in study design, data collection, data analyses, interpretation, or writing of the correspondence.

Supplementary References:

1. He S, Bhatt R, Brown C, Brown EA, Buhr DL, Chantranuvatana K, et al. High-plex imaging of RNA and proteins at subcellular resolution in fixed tissue by spatial molecular imaging. Nature Biotechnology. 2022;40(12):1794-806.

2. Liu N, Bhuva DD, Mohamed A, Bokelund M, Kulasinghe A, Tan Chin W, et al. standR: spatial transcriptomic analysis for GeoMx DSP data. Nucleic Acids Research. 2023.

3. Hao Y, Hao S, Andersen-Nissen E, Mauck WM, 3rd, Zheng S, Butler A, et al. Integrated analysis of multimodal single-cell data. Cell. 2021;184(13):3573-87 e29.

4. Cortal A, Martignetti L, Six E, Rausell A. Gene signature extraction and cell identity recognition at the single-cell level with Cell-ID. Nat Biotechnol. 2021;39(9):1095-102.

5. Foroutan M, Bhuva DD, Lyu R, Horan K, Cursons J, Davis MJ. Single sample scoring of molecular phenotypes. BMC Bioinformatics. 2018;19(1).

6. Aibar S, González-Blas CB, Moerman T, Huynh-Thu VA, Imrichova H, Hulselmans G, et al. SCENIC: single-cell regulatory network inference and clustering. Nature Methods. 2017;14(11):1083-6.

7. Satija. Azimuth Reference - Human Lung (2.0.0). Zenodo. In: Lab S, editor. 2022.

8. Newman AM, Steen CB, Liu CL, Gentles AJ, Chaudhuri AA, Scherer F, et al. Determining cell type abundance and expression from bulk tissues with digital cytometry. Nat Biotechnol. 2019;37(7):773-82.

9. Hahsler M, Piekenbrock M, Doran D. dbscan: Fast Density-Based Clustering with R. Journal of Statistical Software. 2019;91(1).

10. Pliner HA, Shendure J, Trapnell C. Supervised classification enables rapid annotation of cell atlases. Nat Methods. 2019;16(10):983-6.

11. Aegerter H, Lambrecht BN, Jakubzick CV. Biology of lung macrophages in health and disease. Immunity. 2022;55(9):1564-80.

12. Gandolfo LC, Speed TP. RLE plots: Visualizing unwanted variation in high dimensional data. PLOS ONE. 2018;13(2):e0191629.

13. Robinson MD, Oshlack A. A scaling normalization method for differential expression analysis of RNA-seq data. Genome Biology. 2010;11(3):R25.

14. Robinson MD, McCarthy DJ, Smyth GK. edgeR: a Bioconductor package for differential expression analysis of digital gene expression data. Bioinformatics. 2010;26(1):139-40.

15. Ritchie ME, Phipson B, Wu D, Hu Y, Law CW, Shi W, et al. limma powers differential expression analyses for RNA-sequencing and microarray studies. Nucleic Acids Research. 2015;43(7):e47-e.
